# Quality of life and symptom burden in children with neurodegenerative diseases: using PedsQL and SProND, a new symptom-based scale

**DOI:** 10.1186/s13023-022-02485-5

**Published:** 2022-09-02

**Authors:** Annie Ting Gee Chiu, Sheila Suet Na Wong, Naomi Wing Tung Wong, Wilfred Hing Sang Wong, Winnie Wan Yee Tso, Cheuk Wing Fung

**Affiliations:** 1 Department of Paediatrics and Adolescent Medicine, Hong Kong Children’s Hospital, Hong Kong SAR, China; 2 Department of Paediatrics and Adolescent Medicine, Li Ka Shing Faculty of Medicine, Queen Mary Hospital, University of Hong Kong, Hong Kong SAR, China

**Keywords:** Paediatric neurodegenerative diseases, Health-related quality of life, Symptom burden, PedsQL, SProND

## Abstract

**Background:**

Children with neurodegenerative conditions (CNDC) often suffer from severe neurodisability and high symptom burden with multisystemic involvement. However, their symptom burden and health-related quality of life (HRQOL) is not systematically documented in the literature, and there is no existing tool for such purposes. We designed our own tool for scoring of symptom burden amongst CNDCs and adopted the PedsQL generic score 4.0 to quantify the impact of overall symptom burden on children’s overall HRQOL.

**Methods:**

The Symptom Profile for children with neurodegnerative condition (SProND) questionnaire was developed, which consisted of 14 questions grouped according to 5 categories, namely epilepsy, neurobehavioural, movement and mobility related, breathing and swallowing, and other daily activities. CNDCs were recruited during visits to the Comprehensive Neurometabolic / Neurodegenerative Program of the Duchess of Kent Children’s Hospital and Hong Kong Children’s Hospital between November 2019 and March 2020. The SProND and PedsQL 4.0 Generic Core Scales were distributed to consenting parents of CNDCs.

**Results:**

36 CNDCs were recruited and matched with community controls. The response rate of subject and control were 99.5% and 98.7% respectively. The Cronbach alpha was 0.61 for the neurobehavioural domain and >  = 0.7 for other domains. The greater number of symptoms each subject experiences, the worse his/ her PedsQL scores. Subjects displaying hypersalivation and swallowing difficulties had average physical health summary scores of less than 30% compared with subjects without these symptoms. On the other hand, average psychosocial health summary scores of subjects with involuntary movements, joint stiffness, hypersalivation, sleep problem and anorexia were approximately 70% compared to subjects without these symptoms.

**Discussion and conclusion:**

This is one of the first studies to look at CNDCs as a group. We propose the SProND questionnaire for evaluation of symptom profile amongst CNDCs with satisfactory internal and external validity. It demonstrates how physical symptoms impact both physical and psychosocial HRQOL, and the cumulative effect of individual symptoms on the overall HRQOL. As such, CNDCs should be systematically screened for multi-systemic symptoms as a routine part of their clinical care, and care plans should be individually catered to individual patients’ symptom burden and specific needs.

## Background

Paediatric neurodegenerative diseases are a group of disorders characterized by progressive loss of skills and symptoms arising from the central nervous system in children [5] with initially normal or mildly delayed development eg. neuronal ceroid lipofuscinosis and adrenoleukodystrophy, vanishing white matter disease. Children with such conditions suffer from progressive cognitive and physical deterioration and eventually succumb to the course of the disease. Despite the rarity of each of these conditions, collectively they contribute to significant morbidity and mortality within the paediatric population [5, 10].

Children with neurodegenerative conditions (CNDC) often suffer from severe neurodisability and high symptom burden with multisystemic involvement^1^. However, the spectrum and severity of their symptom burden is not systematically documented in the literature, except for specific neurodegenerative conditions. Likewise, the health-related quality of life (HRQOL) amongst children with neurodegenerative condition is not well studied. Available literature pertains to individual diseases with predominantly adult patients, eg. Huntington’s disease [4], Friedrich’s ataxia [3]. Common HRQOL tools such as PedsQL has also not been adopted in CNDCs.

To the best of our knowledge, no prior study has sought to quantify the types and severity of symptoms experienced by CNDCs and their HRQOL in a systematic manner. Whereas generic HRQOL tools like PedsQL may reflect a child’s general health status, they cannot quantify and document the impact of individual symptoms in ways specific to CNDCs. Without such knowledge, it is difficult to ascertain how individual symptoms correlate with the overall health related quality of life amongst CNDCs, and how the medical personnel can best alleviate their symptom burden in a systematic manner, especially during busy clinic consultation with limited time.

This prompted us to design our own tool for documentation and scoring of symptom burden amongst CNDCs. Using this tool, we sought to quantify the impact of overall symptom burden on children’s overall HRQOL. It is hoped that such information may shed light on the experience of these children, and facilitate timely allocation of appropriate attention and services. Such a tool may also be helpful in measuring perceived symptom burden in children undergoing interventional trials in the future.

## Methodology

The Symptom Profile for children with neurodegnerative condition (SProND) questionnaire was developed in English with reference to available literature [7–10]. This was translated into Chinese using back and forth translation by two medical professionals who were fluent in both languages. It consisted of 14 questions, grouped according to 5 categories, namely epilepsy, neurobehavioural, movement and mobility related, breathing and swallowing, and other daily activities. The primary carer is asked to rate the severity of each symptom on a pre-determined scale of one (asymptomatic) to five (worst level of symptom), which were assigned with reference to the authors’ clinical experience and existing literature [11, 13]. As such, the total score on the SProND scale ranges from 14 to 70 (see Table 1).

**Table 1:** Symptom Profile for children with neurodegenerative condition (SProND) questionnaire

	How did the following symptoms affect your child in the past 3 months?				
Seizure	No	< 1x/ month	Monthly (1-3x/ month)	Weekly (1-6x/ week)	Daily (> = 1x/ day)
*Neurobehavioural symptoms*					
Hyperactivity behaviour (Voluntary activities with extreme levels of activity)	No	Minimal	Mild	Moderate	Severe
Aggressive behaviour Eg. biting/ hitting others	No	Minimal	Mild	Moderate	Severe
*Movements and mobility related*					
Involuntary movements	No	Minimal	Mild	Moderate	Severe
Joint stiffness	No	Minimal	Mild	Moderate	Severe
Muscle spasm (Non epileptic sustained muscle contraction)	No	Mild spasms induced by stimulation or exercise	Monthly (1-3x/ month)	Weekly (1-6x/ week)	Daily (> = 1x/ day)
*Breathing and swallowing*					
Swallowing difficulty	No	Full oral feeding with mildly thickened liquid	Full oral feeding with moderately thickened liquid	Partial oral feeding only	Non oral feeding
Need for respiratory support	No	Use of oxygen during viral illness	Use of non-invasive ventilation during viral illness	Nocturnal oxygen/ non invasive ventilation	Whole day usage of O2/ non invasive ventilation or tracheostomy in situ
Drooling	No	Mild and not requiring medication	Tolerable after use of medication	Persistent despite medications, need suction on as needed basis	Require suctioning at least once per day
*Daily activities*					
Pain Site(s): ________ Most severe site: _________ and its severity: mild/ moderate/ severe	No	< 1x/ month	Monthly (1-3x/ month)	Weekly (1-6x/ week)	Daily (> = 1x/ day)
Constipation (Frequency of bowel opening)	1x/ day (average)	> = 2x/ week	Once/ week	< once/ week	< = once/ 2 weeks
Sleep problem Eg. Insomnia, day/night disturbance, night terror etc	No	< 1x/ month	Monthly (1-3x/ month)	Weekly (1-6x/ week)	Daily (> = 1x/ day)
Anorexia/ nausea/ vomiting	No	< 1x/ month	Monthly (1-3x/ month)	Weekly (1-6x/ week)	Daily (> = 1x/ day)
Urinary problem	No	Occasional leakage/ urgency or needing diapers at night	Frequent leakage/ urgency or requiring daytime use of diapers	Requiring intermittent catheterization	Requiring indwelling urinary catheter or nephrostomy

Subjects were recruited from the Comprehensive Neurometabolic / Neurodegenerative Program of the Duchess of Kent Children’s Hospital and Hong Kong Children’s Hospital from November 2019–March 2020. Children aged between 2 and 18 years of age with clinically evident neurodegeneration were included, with or without a confirmed genetic diagnosis. The SProND and PedsQL 4.0 Generic Core Scales [14, 15] a 23-item questionnaire with a total score of 0–100 (higher score represents better quality of life), covering Physical functioning, Emotional functioning, Social functioning and School functioning and validated in Chinese [1], were distributed to consenting parents during clinic visits. Community based age and sex matched control subjects were recruited amongst hospital staff, or through web link containing the relevant questionnaire between April 2020 and June 2021. Each of the subjects was scored according to the Karnofsky/ Lansky Scale (KLS), a 0–100 scale which quantifies performance status in children and adolescents. Such scoring was performed by two authors separately at the time of the questionnaire, and retrospectively at 1 year and 2 years prior to the study with reference to clinical records.

Informed consents were obtained from carers/ guardians prior to questionnaire administration. Ethics approval from the Hong Kong West Cluster/ University of Hong Kong Institutional Review Board was obtained.

The results were analyzed using IBM SPSS statistics version 26 software. Demographic data were expressed in mean, standard deviation, median, number, and percentage when appropriate. The mean difference of the SProND and PedsQL 4.0 generic core scale scores between subjects and controls were analyzed with the paired samples t-test. McNemar's Chi-square test was used to detect the percentage difference of the symptoms between subjects and controls. To validate the internal validity of SProND, the scale internal consistency and Cronbach coefficient alpha of the SProND scale were performed. Scales with the Cronbach’s alpha value more or equal to 0.7 were recognized as reliable. To validate for the external validity of SPRoND, independent-samples t-test was used to screen for specific symptoms with statistically significant association with different domains of PedsQL 4.0 generic core scale i.e. physical, social, emotional and school functioning, as well as physical and psychosocial health summary scores. Pearson correlation coefficient were used to test association between the number of symptoms with one or more PedsQL subscales. Statistical significance was considered for *p* -values less than 0.05 with 2-tailed.

## Results

36 subjects with neurodegenerative conditions were recruited and matched with same number of age and gender matched controls (see Table 2). The diagnosis of subjects included mitochondrial respiratory chain disorders, disorders of neurotransmitters, leukodystrophy, disorders of pyruvate metabolism, disorders of glucose transport, lysosomal disorders, leukodystrophy, developmental and epileptic encephalopathy, undiagnosed neurodegenerative conditions, and others (see Table 3). Mean KLS at time of questionnaire and at 2 years prior were 58.6 and 56.7 respectively. None of the subjects had an improvement in KLS score, with 7 of them (19.4%) a 10 point reduction over the 2 year period.

**Table 2:** Baseline characteristics and scores of subjects and controls

	Control (n = 36)		Subjects (n = 36)		*p*-value
	Mean	SD/%	Mean	SD/%	
*Gender*					
Male	21	58.3%	21	58.3%	N/A
Female	15	41.7%	15	41.7%	N/A
Age	8.6	4.0	9.2	3.9	0.086
*Gross motor function*					
Walk independently	36	100%	19	52.8%	N/A
Walk with support	0	0%	4	11.1%	N/A
Wheelchair bound	0	0%	13	36.1%	N/A
*PedsQL*					
Physical health summary score	88.8	13.6	38.1	26.9	** < 0.001**
Emotional functioning	77.1	16.1	63.5	21.6	**0.01**
Social functioning	84.2	21.7	41.0	22.2	** < 0.001**
School functioning	80.0	16.6	50.1	19.1	** < 0.001**
Psychosocial health summary score	79.6	15.1	52.0	17.8	** < 0.001**
Total PedsQL score	83.7	13.5	46.3	18.5	** < 0.001**
*SProND score*	22.9	3.0	43.7	14.7	** < 0.001**

Bold indicates *P*-value of < 0.05 is considered statistically significant in the current study

**Table 3:** Diagnostic grouping of recruited subjects

Diagnostic category	Total number of subjects
Disorders of neurotransmitter metabolism Aromatic L-amino acid decarboxylase deficiency Succinic semialdehyde dehydrogenase deficiency Guanidine triphosphate cyclohydrocylase (GTPCH) deficiency*	3
Mitochondrial respiratory chain disorders	7
Disorders of pyruvate metabolism Pyruvate dehydrogenase deficiency*	2
Disorders of glucose transport Glucose-1-transporter deficiency*	2
Lysosomal disorders Metachromatic leukodystrophy	1
Leukodystrophy Alexander disease	1
Developmental and epileptic encephalopathy (DEE)	5
Others Congenital disorder of glycosylation type 1a ACTB related dystonia	2
Undiagnosed neurodegenerative conditions	13
Total	36

*Diagnoses regarded as treatable neurometabolic conditions but subject demonstrated neurodegeneration clinically

The response rate of subject and control were 99.5% and 98.7% respectively. The Cronbach alpha was calculated for all subscale domains of the SProND, which was >  = 0.7 for movement and mobility, breathing and swallowing, daily activities subscale, 0.61 fo the neurobehavioural domain, and statistically significant for all of the subscales (see Table 4).

**Table 4:** Internal consistency, reliability and construct validity of SProND categories

Symptom scale	No of items	Cronbach's alpha	Intraclass correlation	95% C.I	*p*-value
Neurobehavioural	2	0.60	0.60	0.12–0.66	**0.004**
Movements and mobility related	3	0.87	0.87	0.77–0.93	** < 0.001**
Breathing and swallowing	3	0.85	0.85	0.73–0.92	** < 0.001**
Daily activities	6	0.70	0.67	0.48–0.81	** < 0.001**
Total symptom score	14	0.86	0.85	0.76–0.91	** < 0.001**

The PedsQL and SProND scores of subjects and controls differed significantly (see Table 2). Subjects’ mean total PedsQL score was 46.3, approximately half of their age and sex matched controls only. Whereas the SProND had a baseline score of 14, subjects had a mean score of up to 43.7, whilst that of control was only 22.9 (see Table 5).

The presence of all movement and mobility, breathing and swallowing and activities of daily living related symptoms were significantly associated with worse physical health summary score. In particular, subjects displaying hypersalivation and swallowing difficulties had average physical health summary score of less than 30%compared with subjects without these symptoms, with p value of < 0.05. The impact on psychosocial health summary score was less drastic. Subjects with involuntary movements, joint stiffness, hypersalivation, sleep problem and anorexia were associated with statistically lower mean psychosocial health summary score, at approximately 70% of subjects without these symptoms (see Table 5).

**Table 5:** PedsQL score of subjects with and without individual symptoms listed on SProND

	Physical health summary score			Psychosocial health summary score												Total PedsQL score		
				Emotional functioning			Social functioning			School functioning			Total					
	mean score			mean score			mean score			mean score			mean score			mean score		
	Yes	No	*p*-value	Yes	No	*p*-value	Yes	No	*p*-value	Yes	No	*p*-value	Yes	No	*p*-value	Yes	No	*p*-value
1. Seizure (n = 13)	31.3	42.0	0.256	55.4	68.0	0.092	35.4	44.1	0.261	43.9	53.9	0.133	45.0	55.9	0.078	39.8	49.9	0.116
*Neurobehavioural*																		
2. Hyperactivity behaviour (n = 13)	45.4	34.0	0.225	58.5	66.3	0.302	37.3	43.0	0.464	46.7	52.1	0.353	47.6	54.3	0.233	46.5	46.2	0.961
3. Aggressive behaviour (n = 11)	50.6	32.6	0.064	59.1	65.4	0.428	37.3	42.6	0.514	47.2	51.3	0.573	47.5	53.4	0.378	48.5	45.3	0.634
*Movements and mobility related*																		
4. Involuntary movements (n = 26)	32.5	52.8	**0.040**	57.3	79.5	**0.004**	36.9	51.5	0.077	45.1	63.9	**0.008**	46.8*	65.3	**0.005**	40.9	60.2	**0.004**
5. Joint stiffness (n = 23)	24.3	62.5	** < 0.001**	57.8	73.5	**0.035**	34.1	53.1	**0.012**	42.6	62.1	**0.002**	44.9*	62.6	**0.003**	37.3	62.3	** < 0.001**
6. Muscle spasm (n = 19)	24.0	53.9	** < 0.001**	56.3	71.5	**0.034**	36.8	45.6	0.243	45.8	54.8	0.170	46.8	57.2	0.083	37.9	55.7	**0.003**
*Breathing and swallowing*																		
7. Swallowing difficulty (n = 9)	11.8**	46.9	** < 0.001**	55.0	66.3	0.178	31.7	44.1	0.148	40.0	53.1	0.084	43.0	54.4	0.113	30.8	51.4	** < 0.001**
8. Need for respiratory support (n = 6)	17.7	42.2	**0.040**	53.3	65.5	0.213	34.2	42.3	0.599	37.0	52.3	0.094	42.7	53.3	0.219	32.8	49.0	**0.049**
9. Drooling (n = 15)	15.0**	52.8	** < 0.001**	53.2	70.0	**0.012**	29.6	48.2	**0.012**	40.4	55.3	**0.025**	40.7*	57.7	**0.005**	31.3	55.8	** < 0.001**
*Daily activities*																		
10. Pain (n = 9)	22.6	43.3	**0.044**	55.6	66.1	0.209	33.3	43.5	0.238	38.8	53.5	0.051	43.3	54.3	0.126	35.3	50.0	**0.038**
11. Constipation (大便次數) (n = 18)	25.9	50.3	**0.005**	56.9	70.0	0.070	37.5	44.4	0.355	49.1	51.0	0.778	48.3	55.1	0.272	39.4	53.1	**0.024**
12. Sleep problem eg. insomnia, day/ night disturbance, night terror etc.(n = 15)	25.0	47.5	**0.011**	49.7	73.3	**0.001**	31.0	48.1	**0.020**	39.3	57.6	**0.004**	40.2*	59.7	**0.001**	34.6	54.7	**0.001**
13. Anorexia/ nausea/ vomiting (n = 11)	24.4	44.1	**0.041**	53.2	68.0	0.057	32.7	44.6	0.141	40.0	54.2	**0.043**	42.6*	55.5	**0.050**	35.2	51.2	**0.015**
14. Urinary problem (n = 20)	26.3	51.3	**0.004**	56.6	71.2	**0.041**	35.8	46.8	0.140	50.1	50.0	0.988	47.4	56.0	0.153	39.3	54.1	**0.015**

*Mean psychosocial health summary score of subjects with these symptoms is approximately 70% of subjects without these symptoms

**Mean physical health summary score of subjects with these symptoms is less than 30% of subjects without these symptoms

**Fig. 1:**
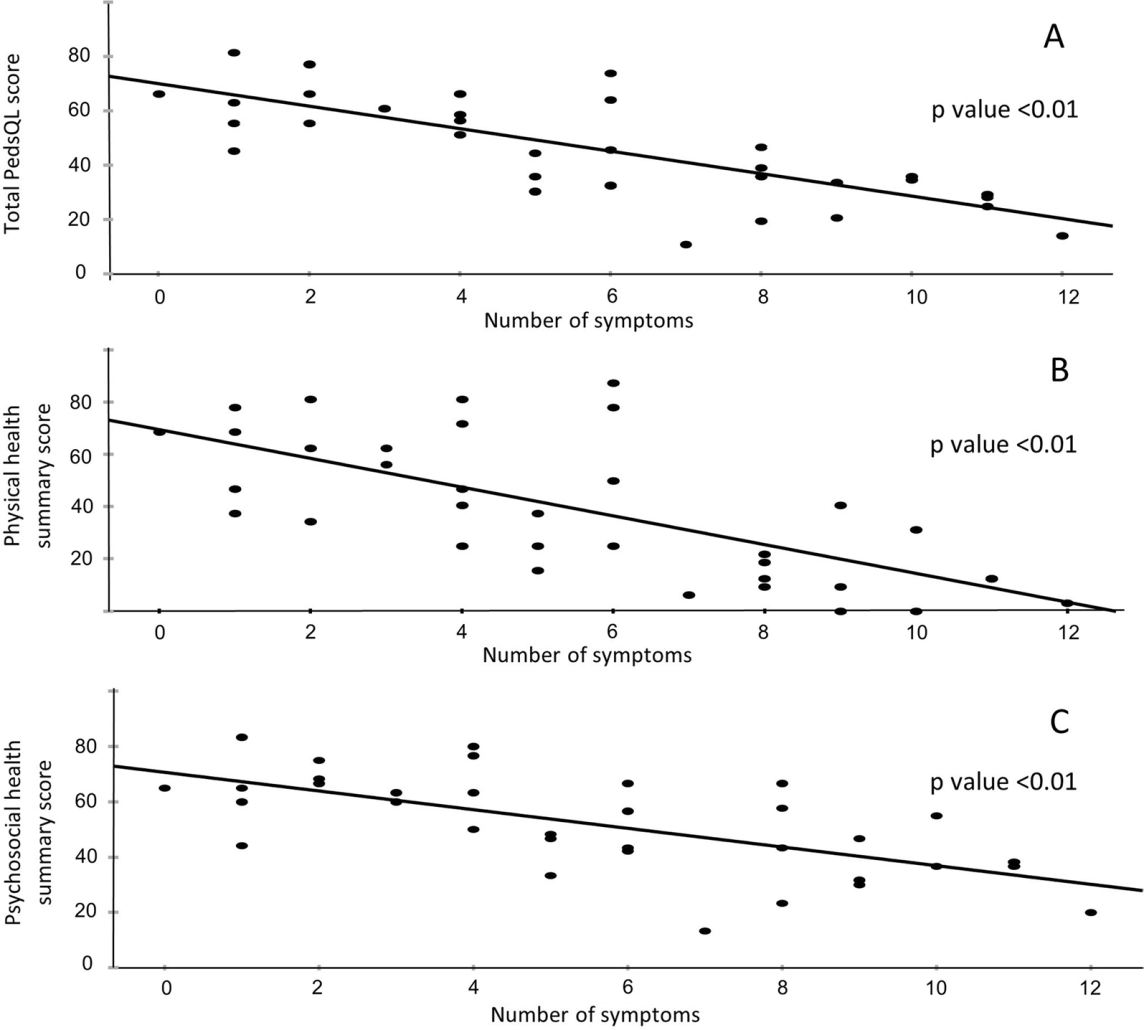
Scatter plots showing negative correlation between number of symptoms and total PedsQL score (**A**), physical health summary score (**B**), psychosocial health summary score (**C**)

**Fig. 2:**
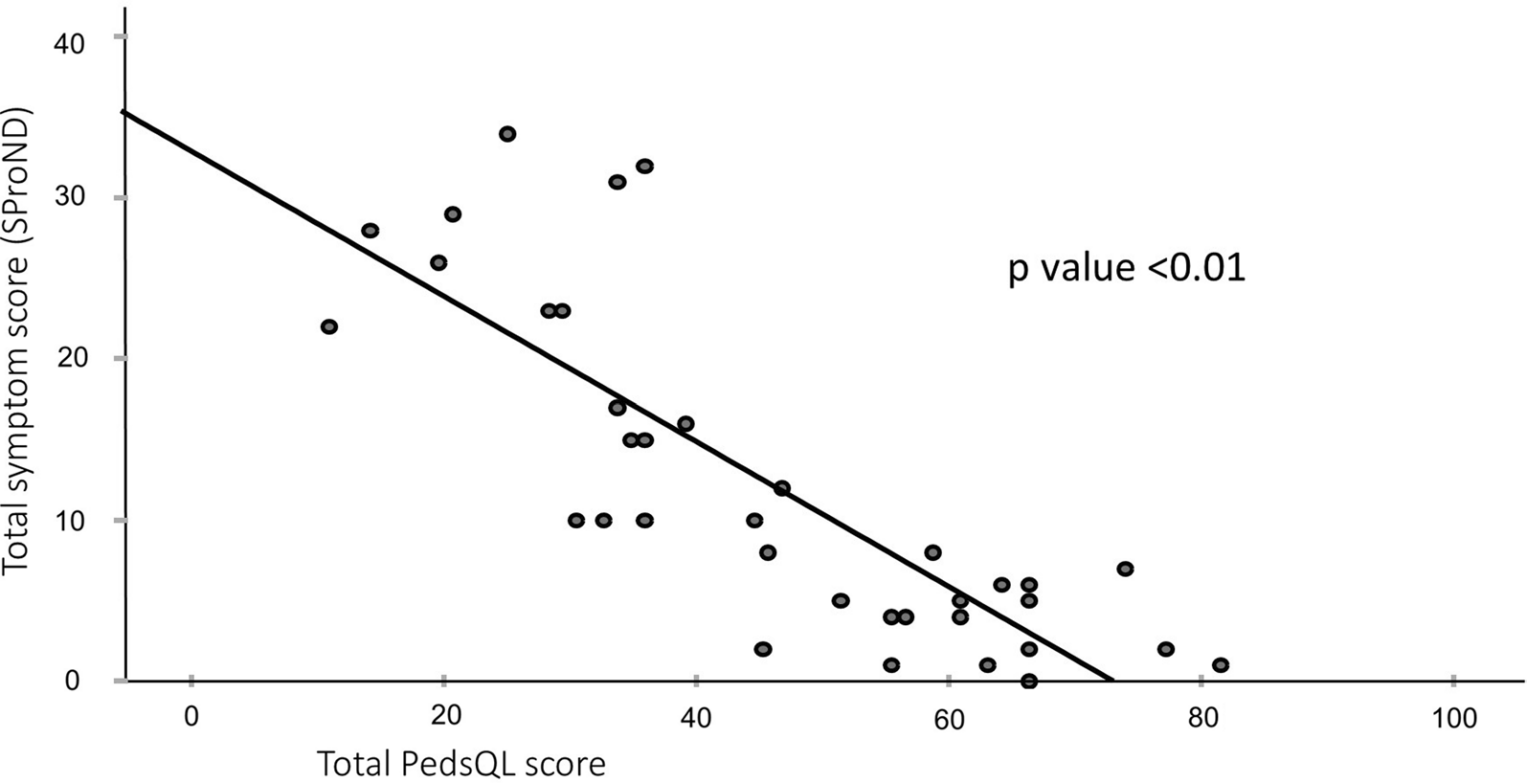
Scatter plot showing negative correlation between SProND and total PedsQL score

Further analysis showed that, the greater number of symptoms each subject experience, the worse his/ her PedsQL scores, which holds true for both physical and psychosocial summary health scores (see Fig. 1). Although the number of subjects precludes meaningful analysis using the severity of individual symptom, the severity integrated SProND score also showed a negative correlation with PedsQL score (see Fig. 2). Comparison of HRQOL and SProND scores between diagnosed and undiagnosed groups did not show any significant difference between the two groups (see Table 6).

**Table 6:** Comparing the PedsQL and SProND scores of diagnosed and undiagnosed subjects

Parameters	undiagnosed patients(= 10)		diagnosed patients (n = 26)		*p*-value
	Mean	SD/%	Mean	SD/%	
*PedsQL(Parent)*					
Physical functioning/Physical health summary score	35.3	23.5	39.2	28.5	0.705
Emotional functioning	66.0	19.6	62.5	22.6	0.670
Social functioning	44.0	22.8	39.8	22.2	0.618
School functioning	50.5	20.5	49.9	18.6	0.930
Psychosocial health summary score	53.5	16.9	50.9	18.2	0.705
Total PedsQL score	50.9	18.2	47.2	17.2	0.861
*SProND Score*	41.8	11.1	45.9	15.9	0.460

## Discussion

This is one of the first studies to look at CNDCs as a group rather than in terms of individual conditions. We propose the SProND questionnaire, a simple 14-item scale for evaluation of symptom profile amongst CNDCs with satisfactory internal and external validity. Overall, this study demonstrates how different individual physical symptoms impact on health-related quality of life amongst children with neurodegenerative conditions, both physically and psychosocially. The mean score of subjects was 46.3. When benchmarked with a local study conducted amongst children with special education needs during the recent COVID-19 pandemic, this falls within the lowest 10% of the SEN population [15].

Contrary to previous studies amongst mucopolysaccharidosis and Batten’s disease, where behavioural symptoms were reported to be more challenging [10] and difficult to manage [2], the impact of behavioural symptoms such as hyperactivity and aggressive behaviour on HRQOL were not statistically significant in this study. The discrepancy between our current findings and that of previous studies highlights that symptom profile can vary greatly even amongst children with different causes of neurodisability. This is also one of the reasons why the neurobehavioural domain was not removed from the scale despite a relatively low Cronbach alpha with neurobehavioural domain.

Whilst there was no significant difference in HRQOL and symptom burden between diagnosed and undiagnosed subjects, physical symptoms impact heavily not only on physical HRQOL but also psychosocial HRQOL, the most notable of these being involuntary movements, joint stiffness, hypersalivation, sleep problem and anorexia. Such result reinforces that care plans should be individually catered to the patients’ needs, and that children with undiagnosed neurodegenerative conditions and be given similar care and attention as those with known specific diagnosis. This may be achieved with use of the proforma developed for this study, which incorporates different symptoms and their severity, and may aid clinicians in monitoring the symptom profile of different patients over time.

Although symptom-related impact is often a subjective experience and qualitative analysis is often applied, quantitative analysis adopted in this study helped to demonstrate the cumulative effect of individual symptoms on the overall HRQOL. From a healthcare planning point of view, the findings of this study support the allocating appropriate resources to children with disabilities according to the severity of their symptoms, rather than in an all-or-none manner. CNDCs should also be systematically screened for disturbing symptoms to ensure holistic care of the patients.

The main limitation of the study was its sample size, which precluded further analysis based on individual symptoms owing to the small sample size. Looking forward, it will be important to expand the cohort for improved statistical power, which would allow more in-depth analysis and potentially generate results of wider impact. It will also be interesting to follow up CNDCs in a longitudinal manner to ascertain the evolution of their symptoms with disease progression, and how repeated losses experienced by these parents [6] interplay with carer stress and reporting of symptom severity, as stressful families tend to report greater symptoms [12]. SProND may also be adopted in future clinical trials for better gauging of subjects’ symptom burden. Furthermore, the correlation between symptom severity and PedsQL scores identified in the study opens future possibilities to calculate Quality Adjusted Life Years (QALY) based on symptomatology and disease trajectory, which will be especially useful for conditions whose rarity makes it difficult to conduct formal health economic studies.

## Conclusion

CNDCs suffer from high symptom burden, including behavioural and physical symptoms which impact significantly on their physical and psychosocial quality of life. CNDCs should be systematically screened for multi-systemic symptoms as a routine part of their clinical care.

## Data Availability

Relevant data and materials are available upon request to corresponding authors.

## Abbreviations

CNDC: Children with neurodegenerative conditions
HRQOL: Health-related quality of life
SProND: Symptom profile for children with neurodegnerative conditions
KLS: Karnofsky/ Lansky Scale
